# Exploring the Link between Evening Chronotypes and T2DM among South Indians: A Cross-Sectional Study

**DOI:** 10.2174/0115733998406914251104020940

**Published:** 2026-01-15

**Authors:** Meghana Belagula Siddaraju, Akalu Srinivas Kamakshi Shriya, Sri Ram Murugesh, Varun Thej Reddy, Arun Gopi, Nimrah Fathima

**Affiliations:** 1 General Medicine, JSS Medical College & Hospital, Mahatma Gandhi Road, Mysuru, 570004, Karnataka, India;; 2 Pharmacy Practice, JSS College of Pharmacy, JSS Academy of Higher Education and Research, Mysuru, 570015, Karnataka, India;; 3 Community Medicine, JSS Medical College, JSS Academy of Higher Education and Research, Mysuru 570015, Karnataka, India

**Keywords:** Diabetes mellitus, chronotype, morningness-eveningness questionnaire, BMI, waist to hip ratio, glycemic control

## Abstract

**Introduction:**

Chronotype refers to an individual's circadian preference in biological and behavioral rhythm relative to the external light-dark cycle. light-dark cycle. Evening chronotype is associated with increased diabetes risk, probably due to inappropriate eating habits, reduced physical activity, and altered sleeping habits. This study aims to find an association between evening chronotropic and diabetes.

**Methods:**

A cross-sectional study was conducted in the General Medicine department over six months. 201 patients satisfying the study criteria were included. Horne-Ostberg Morningness-Eveningness Questionnaire (MEQ), blood samples, sleep quality, and anthropometric indicators were obtained. Postprandial blood sugar (PPBS), fasting blood sugar (FBS), glycated hemoglobin (HbA1c), Total cholesterol (TC), triglycerides (TGs), High-density lipoprotein cholesterol (HDL-C), low-density lipoprotein cholesterol (LDL-C), and very low-density lipoproteins (VLDL) were measured with standard techniques. A univariate logistic regression analysis, along with post-hoc tests, was employed to find the role of independent variables on each chronotype.

**Results:**

Among the 81 evening chronotypes, 85.1% were diabetics, the mean HDL value in the evening chronotypes was 35.2 mg/dl, significantly (*p*<0.001) lower than the other two chronotypes. Waist-to-hip ratio (WHR) and body mass index (BMI) were significantly higher in the evening chronotype *p*= 0.011, *p*<0.001, respectively. The mean FBS, PPBS, and HbA1c values (170.07 mg/dL, 242 mg/dL, and 8.95%) were higher among evening chronotypes and were statistically significant (*p*<0.001).

**Discussion:**

Findings highlight that evening chronotypes demonstrate poorer glycaemic control, dyslipidaemias, and adverse anthropometric indices, suggesting that circadian misalignment may contribute to T2DM progression. Lifestyle modification could be a promising intervention.

**Conclusion:**

Our study suggests Evening chronotypes are more likely to have metabolic dysregulation and poor glycemic control.

## 1 INTRODUCTION

Type 2 diabetes mellitus (T2DM) is a metabolic condition characterized by chronic high blood sugar levels caused by either insulin resistance, insufficient insulin secretion, or a combination of both [1, 2]. Approximately 537 million adults between the ages of 20 and 79 live with T2DM globally, representing 10.5% of this age group. It is forecasted that by 2030, the number of individuals with diabetes worldwide will reach 643 million, increasing to 783 million by 2045 [3, 4]. India is expected to have 134 million diabetics by 2045, according to the International Diabetes Federation (IDF) [5-7]. Chronotype refers to a characteristic that indicates a person's circadian preferences related to behavioral and biological rhythms in relation to the external light-dark cycle [8, 9]. This classification distinctly categorizes individuals based on their preferred sleep habits, with morning types going to bed early and waking with the sunrise, and evening types tending to stay awake late and rise later. In contrast, intermediate types show a lack of strong preference for either schedule. The circadian rhythm is recognized for its influence on glucose metabolism [10, 11]. Research has shown that individuals with an evening chronotype exhibit a preference for certain behavioral and biological patterns that correspond with increased glucose tolerance and elevated inflammatory markers, thereby heightening cardiometabolic risk [12, 13]. Evening chronotype is linked to a greater risk of T2DM, likely due to lower levels of physical activity, unhealthy eating habits during late hours, and disrupted sleep patterns [14-16]. Both duration and quality of sleep significantly impact blood glucose regulation in individuals with T2DM [17-19]. An evening chronotype, along with larger dinner sizes, has been correlated with poorer glycaemic control in T2DM patients [20-23]. These findings indicate that an individual's chronotype may predict disease outcomes and further underscore the circadian system's role in metabolic regulation. As far as the authors are aware, only one study by Suneeta S. Chandorkar *et al.* investigates the link between chronotype and glycaemic control within the Indian population [24]. Although some systematic reviews have been conducted by Indian researchers on studies from other regions [25, 26]. The study aims to find if there is an actual association between evening chronotropic and diabetes risk, and to enlighten the new approaches and targets for interventions addressing lifestyle changes to combat the rising trends in diabetes mellitus.

## 2 METHODS

### 2.1 Study Design and Ethical Clearance

A cross-sectional study was carried out in the Department of General Medicine between January 2024 and June 2024 at a tertiary care hospital in South India. Before the study commenced, approval was obtained from the local institutional ethics committee (IEC) (JSSMC/IEC/070324/52 NCT/2023-24), and the Informed consent forms (ICFs) were designed as per the IEC requirements.

### 2.2 Sample Size

The sample size was calculated based on the prevalence of diabetes in the Indian population (*p*=11.4%; 0.114), as reported in a study by Ranjit Mohan Anjana *et al. *[27]. Q, calculated using the formula 1-P (Q=0.886). Additionally, the confidence interval was set at 5% (D=0.05), and the Z score at the 0.05 level was observed to be 1.96. The required number of samples (n) was determined to be 155. However, a total of 201 patients were recruited for the study due to greater availability.

S= Z^2^PQ/D^2^

### 2.3 Sampling Methods

After checking the inclusion and exclusion criteria, a convenience sampling method was used to select the study participants.

### 2.4 Study Criteria and Flow

Patients above 18 years of age with or without T2DM and who could read/ understand Kannada (local language)/ English after giving informed consent were included in the study. Patients with T1DM, gestational diabetes, drug-induced diabetes, shift workers with sleep disorders, insomnia medication recipients, and newly diagnosed T2DM patients within the past six months were excluded from the study. Patients meeting the study criteria were included in the research.

The Horne-Ostberg MEQ was used to categorize study participants into morning, evening, or intermediate chronotypes [28]. The MEQ consists of 19 straightforward questions regarding the daily performance and desired sleep schedules. Additionally, respondents were required to provide information regarding their dinner time, bedtime, sleep duration, and midnight snacking habits. We assessed the quality of sleep based on participants' experience and expressed it as good, moderate, or poor. The STROBE diagram is represented in (Fig. **1**).

The participants' anthropometric measures were measured in accordance with the standards of the International Diabetes Federation [29]. These measures were weight (in kilograms), height (in centimeters), hip circumference, and waist circumference (in centimeters). Weight in kilograms divided by height in square meters was used to compute BMI. A normal WHR was defined as 0.9 or less for men and 0.8 or less for women. WHR was computed by dividing WC by hip circumference.

Additionally, blood samples were collected at the same visit to measure Total cholesterol (TC), HDL-C and LDL-C, TG, VLDL-C, FBS, PPBS, and HbA1C. Lipid parameters and FBS were measured from a fasting blood sample.

### 2.5 Tools Used for the Study

Utilized data collection forms (DCFs) which were explicitly designed to capture relevant information such as age, time of dinner, time of sleep, duration of sleep, midnight snacking, duration of DM, FBS, PPBS, HbA1C, BMI, TC, TG, HDL, LDL, VLDL, and MEQ score.

Lipid profile tests such as HDL, LDL, TG, and TC were assessed utilizing the polymer polyanion method, direct measure method, enzymatic endpoint method, and cholesterol peroxidase method, respectively, in the Roche Cobas pure instrument (under the manufacturer's protocol). Additionally, VLDL was calculated using the formula TG/5 mg/dl. We measured Glucose tests such as FBS and PPBS using the enzymatic electrochemical detection method in Accu-Chek glucometers and HbA1C using a high-performance liquid chromatography method in a Bio-Rad D-10 machine (following the manufacturer's protocol). All instruments were internally calibrated daily at the start, and additional external calibrations were conducted monthly.

### 2.6 Definitions

Morning chronotypes: Individuals with the Horne Ostebergs MEQ score of 59-86 were classified as morning chronotypes [30].

Intermediate chronotypes: Individuals with the Horne Ostebergs MEQ score of 42-58 were classified as intermediate chronotypes [30].

Evening chronotypes: Individuals with the Horne Ostebergs MEQ score of 16-41 were classified as evening chronotypes [30].

### 2.7 Statistical Analysis

The IBM Corp. (2013) IBM SPSS Statistics for Windows, Version 22.0. IBM Corp., Armonk, NY, was used to carefully examine the data gathered from the questionnaire after it was methodically entered into Microsoft Excel version 19. The arithmetic mean and standard deviation (SD) have been used to depict the demographic information, anthropometric measurements, lipid profiles, and blood sugar testing. However, both the arithmetic means and SD, as well as numbers and percentages, were used to depict the snacking and sleeping profiles. The comparison of quantitative variables such as age, HDL, LDL, *etc.*, across three groups, namely morning, intermediate, and evening chronotypes, has been done using an ANOVA followed by a post hoc Bonferroni analysis wherever required. A Chi-square test was additionally used to analyze categorical variables, such as gender, sleep quality, *etc.*, across the three chronotypes. Univariate and multiple logistic regression analyses have been done to find the potential association between the diabetic risk and the chronotypes. *p* =<0.05 is regarded as statistically significant.

## 3 RESULTS

A total of 201 individuals who met the criteria for the study were recruited. According to the MEQ score, the participants were categorized into morning, intermediate, and evening chronotypes. The MEQ results clearly show that 78 patients belong to the morning chronotype, 42 to the intermediate chronotype, and 81 to the evening chronotype. One hundred patients gave a history of T2DM and were on treatment for the same. Among the 81 evening chronotypes, 85.1% (69) were diabetics and 14.9% (12) were non-diabetics, suggesting more diabetics have evening preference (Fig. **2**).

The mean age among the evening chronotypes was 51.69 years, and 31.65 years in the morning type. In evening chronotypes, 44 (54.2%) were females. We compared the different metabolic parameters among the chronotypes. The mean HDL value in the evening chronotypes was 35.2 mg/dl, significantly (*p*<0.001) lower than the other two chronotypes. However, we found no significant difference in other lipid parameters (*p*>0.005). Individuals with an evening chronotype exhibited significantly higher BMI and WHR, *p* =<0.001. The average BMI for this group was 27.76 kg/m^2^, and their WHR was 0.82, greater than that of the other chronotypes. The mean values for FBS, PPBS, and HbA1c were found to be higher among individuals with evening chronotypes, with values of 170.07 mg/dL, 242mg/dL, and 8.95%, respectively. The differences observed were statistically significant, with a *p*-value of less than 0.001. Also, individuals with intermediate chronotypes had higher FBS, PPBS, and HbA1c values (142.40 mg/ dL, 207.71mg/ dL, and 8.09%) compared to those with morning chronotypes, who recorded values of 98.38 mg/ dL, 132.46mg/ dL, and 5.61%. This result was also statistically significant, *p* =< 0.001. The association of the characters influencing diabetes is represented in Table **1**.

The analysis reveals a significant relationship between chronotype and sleep quality. Observations portray that the majority of the morning chronotype individuals reported “good” sleep quality, with a small percentage experiencing “moderate” sleep, and no cases of “poor” sleep were observed. Evening chronotypes showed a different pattern, with a minority reporting “good” sleep, while higher proportions experienced “moderate” and “poor” sleep quality. Intermediate chronotypes demonstrated the most diverse distribution, with a majority reporting “good” sleep but notable proportions also experiencing “moderate” and “poor” sleep quality. The chi-square test confirmed that the distribution of sleep quality varies significantly across chronotypes, with a *p* = < 0.001.

In summary, sleep quality is closely associated with chronotype, with morning chronotypes generally experiencing better sleep quality than evening and intermediate chronotypes. Conversely, there were no notable variations in sleep length, sleeping hours, late-night snacking, and dinner times between the 'morning type' and the 'evening type' groups. (Table **2**) portrays the sleep profiles and snacking patterns among the chronotypes. Further, Table **3** depicts the Univariate and multiple logistic regression, along with the odds ratio to find the potential association of diabetes risk and chronotypes. Intermediate chronotype showed no significant association with diabetes in either univariate or multiple logistic regression analyses. However, Evening chronotype was significantly associated with increased odds of diabetes in both univariate and multiple logistic regression models (*p*<0.05).

## 4 DISCUSSION

In our study, we reported that 85.1% of the patients were diabetics among evening chronotypes, which means they were active during the later period of the day, as opposed to the morning chronotype. While 28.7% were diabetics in the evening chronotype compared to 20.5% in the morning chronotype in a study done by Yu *et al.*, most of the studies did not have a non-diabetic control group [13]. In evening chronotype individuals, dyssynchrony in the internal circadian rhythm is regulated by the suprachiasmatic nuclei, with the external circadian rhythm regulated by light exposure [31-35]. There are many studies to prove that the disturbance in the above circadian rhythm elevates the inflammation in the body, causing an increase in the high-sensitivity C-reactive protein (hs CRP levels), decreased insulin sensitivity, impaired glucose tolerance, and other metabolic derangements [36-38].

A study by Henson J *et al.* proved that physical behaviors across chronotypes were distinct. Higher sedentary time (28.7 min/day, 95% CI 8.6 to 48.3) was observed in the lifestyle of evening chronotypes as compared to other types. This was assessed utilizing an accelerometry device, which assesses moderate to vigorous physical activity (MVPA) and quantifies physical activity over a period of time. The author also stressed the fact that there was a lower MVPA, reduced light activity, along with decreased intensity observed among evening chronotypes [39].

A study done by Iwasaki *et al.* [40] observed that there is poor control of diabetes in evening chronotype patients, which is consistent with the current study with significantly raised HbA1C levels 8.3%± 1.8% *vs.* 8.95% ± 2.59%, respectively, same was supported by Hashemipour *et al.* [41]. Also, the HDL levels in the evening chronotypes were negatively correlated with the MEQ scores when compared to the morning cohort, similar to our study 47.4 mg/ dL ± 9.1 mg/ dL, 55.1 ± 12.7 *vs.* 35.21 mg/ dL ± 9.16 mg/ dL, 42.09± 8.07.

Similar to the study done by Chandorkar *et al.* [24], the FBS and PPBS levels in our study were higher in evening chronotypes. It is a well-known fact that sleep duration and quality of sleep will lead to abnormal glucose metabolism [42]. Evening chronotype type patients tend to sleep late and get up early to conform to their social obligations, thus reducing the sleep duration and hampering the sleep quality [43-51]. Though sleep quality was significantly poor in the evening cohort, the sleep duration failed to show any link to diabetes control, proving the possible involvement of other factors. This point is proved by a few studies where night shift workers suffer from overall metabolic mortality and morbidity with increased insulin resistance, hyperleptinemia, high blood pressure and disrupted cortisol rhythms occur in night shift workers resulting in obesity, diabetes [52], metabolic syndrome, cardiovascular and gastric disorders [53-56] with increased risk related to number of shifts per month [57].

The patients in the morning chronotype tend to consume their dinner early and sleep earlier compared to the evening chronotype, thus reducing midnight snacking or less total daily energy intake [58], and completing good hours of sleep. Whether these habits have contributed to the metabolism leading to significantly reduced T2DM patients in this cohort is still under scrutiny. Only chronotype has been shown to have a notable role irrespective of food intake and sleep duration in a few studies [59]. The insulin secretion rate is maximum between 12: 00 hours and 18: 00 hours and at its lowest from midnight to 6:00 hours [60] which is inversely proportional to the melatonin secretion, a circadian rhythm marker [61]. So having dinner early helps patients to improve their glucose metabolism; however, the exact mechanism is not known [61-64]. In line with the above statement, treatment with melatonin before bed in 60 patients with coronary heart disease and diabetes for three months reduced systemic inflammation in a study done by Raygan *et al.*, [65]. Though its advantage across chronotypes is unclear.

The above study observed that 14.9% of patients who were non-diabetics in evening chronotypes had high levels of FBS and PPBS, suggesting impaired glucose metabolism. Healthy individuals tend to develop all the mentioned adverse effects of sleep disturbance and misaligned circadian rhythm, and in the long run, can develop metabolic syndrome. This can be concluded by conducting a prospective study and long-term follow-up of such patients, which might help to derive a causal relationship. Anothaisintawee *et al.* proved a similar view in patients with prediabetes, where they concluded that irrespective of sleep disturbances and social jetlag (discrepancy between the circadian clock and social rhythms), patients with prediabetes with evening chronotypes had higher HbA1c levels (5.83%, *p* = 0.019, 95% CI: 0.00001, 0.038, *p* = 0.049) [66]. Few studies showed association of social jetlag and development of T2DM [67-69], obesity, lipid metabolism [70], cancer and mental health diseases [71].

Not limited to glucose metabolism, evening chronotypes had increased all-cause mortality when compared to morning types after 6.5 years of follow-up in the UK Biobank cohort analysis. Hence, effective nonpharmacological therapy can be incorporated into the lifestyles of individuals by being more active during the early hours of the day to be more synchronous with the circadian rhythm to finish their dinner early, sleep early with fewer disturbances at night, having good quality sleep which can help them to reduce the inflammation in the body and have better control of diabetes [72-74]. This seems plausible with patients with preserved pancreatic function and a shorter duration of diabetes, and is helpful in prediabetes and healthy people with high blood sugar values.

## 5 LIMITATIONS

The current study has its limitations. Firstly, the socioeconomic status, calorie intake, physical activity profile, and light exposure during the night were not recorded, which are known influence the metabolic profile. Secondly, the limited number of available covariates may have influenced this study's outcome due to the effect of unknown confounders. This study had a small sample size from a single center, and it did not include prospective follow-up. This data cannot be generalized; however, it represents the findings observed at our center. Additionally, we did not utilize any scale to assess the quality of sleep in the study population. Though melatonin influences insulin secretion through receptors on pancreatic beta cells and affects glucose metabolism [63], our study is limited by a lack of information on melatonin levels. Future studies can focus on the influence of chronotype on prediabetics and the impact of chronotherapy by prospectively following up the participants

## CONCLUSION

In summary, the evening chronotype population is linked to a higher risk of diabetes and poorer diabetic control, highlighting the significance of circadian alignment in glucose metabolism. While a causal relationship between metabolic syndrome and chronotypes may not be established, given the study type and sample size, this sets the stage for further research. Future studies are required on longitudinal or interventional designs, incorporating adjusted sleep and dinner timings, reduced light exposure at night, and assessments of diabetes control and other metabolic conditions. Thus, providing more conclusive evidence on whether evening chronotype is a modifiable risk factor for T2DM and metabolic syndrome.

## Figures and Tables

**Fig. (1):**
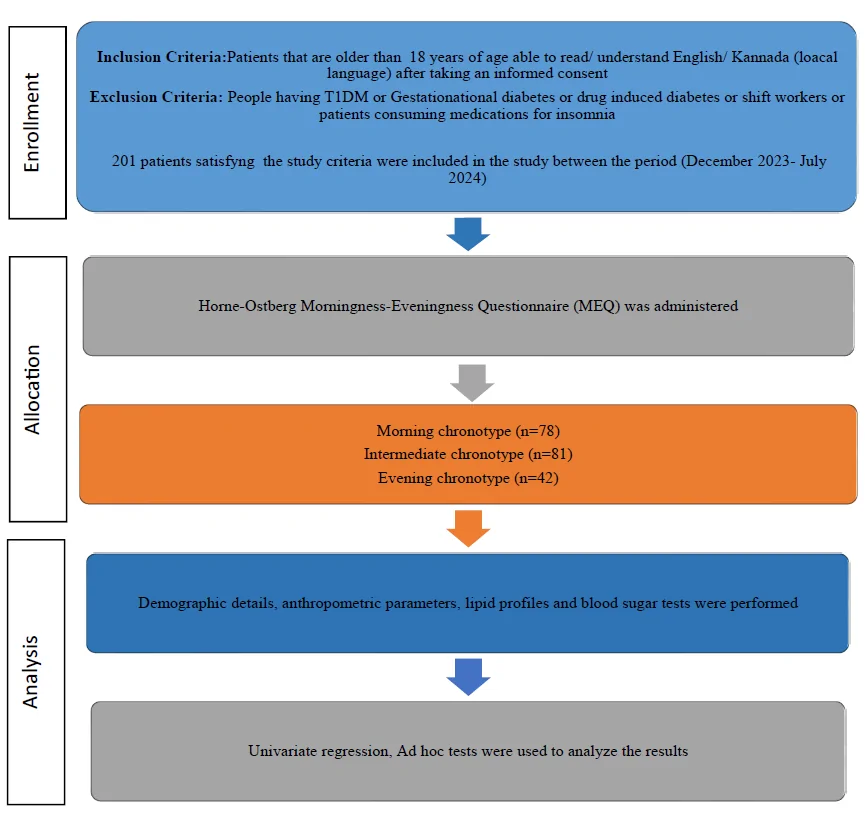
STROBE flow diagram illustrating patient enrollment, allocation, and analysis process.

**Fig. (2):**
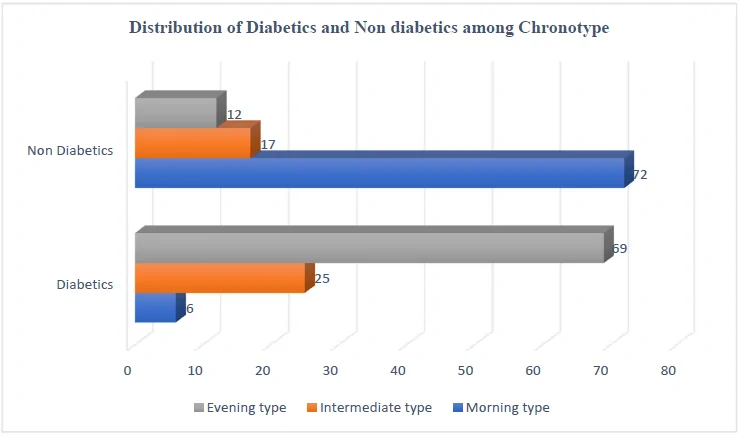
Distribution of diabetes and non diabetics among chronotype.

**Table 1:** ANOVA and post hoc tests of the characteristics of the study participants among the chronotypes.

**Characteristics**	**Morning Type**	**Intermediate Type**	**Evening Type**	***P***
**N**	78	42	81	
**Age, y**	31.65 ± 10.27	46.97± 9.93 ^a^	51.69± 8.90 ^b^	<0.001
**Gender, female**	36 (46.15%)	19 (45.23%)	44 (54.32%)	0.496
**Gender, male**	42 (53.84%)	23 (54.77%)	37 (45.68%)	
**TC, mg/dL**	152.56 ±40.78	148.00± 55.77	143.24± 46.47	0.452
**HDL, mg/dL**	42.09± 8.07	34.24± 12.01 ^a^	35.21± 9.16 ^b^	<0.001
**LDL, mg/dL**	91.67± 35.17	93.44± 44.34	92.04± 44.10	0.974
**VLDL, mg/dL**	28.75± 15.07	33.82± 20.37	31.10± 19.07	0.33
**TG, mg/dL**	145. 69± 75.19	169.10± 101.86	155.31± 95.17	0.392
**BMI, kg/m^2^**	24.51± 4.66	25.62± 4.40	27.76± 5.48 ^b^	<0.001
**Waist/ hip ratio**	0.78± 0.07	0.81± 0.08	0.82± 0.09 ^b^	0.011
**MEQ score, total**	69.83± 5.99	55.38± 12.48 ^a^	36.21± 2.99 ^b^	<0.001
**Duration of DM, y**	0.37± 1.55	4.47± 6.45 ^a^	6.15± 6.02 ^b^	<0.001
**FBS, mg/dL**	98.38± 29.63	142. 40± 56. 90 ^a^	170.07± 65.78 ^b^	<0.001
**PPBS, mg/dL**	132.46± 40.58	207.71± 80.35 ^a^	242± 81.13 ^b^	<0.001
**HBA1C, %**	5.61± 1.00	8.09± 2.78 ^a^	8.95± 2.59 ^b^	<0.001

^a^ *p*<0.05 *vs.* morning type; ^b^*p*<0.05 *vs.* intermediate type; HbA1C: Glycated Haemoglobin; FBS: Fasting blood sugar; PPBS: Postprandial blood sugar; MEQ: Morningness-Eveningness Questionnaire; BMI: Body mass index; TG: Triglyceride; TC: Total cholesterol; LDL: low-density lipoprotein; HDL: High-density lipoprotein; VLDL: very low-density low protein; y: years.

Data depicted as number (percentage); mean± std deviation.

**Table 2:** Sleep profiles and snacking habits based on chronotype.

**Sleep Profiles**	**Morning Chronotype**	**Intermediate Chronotype**	**Evening Chronotype**	***P***
**Sleep duration, hours**	8.09± 1.11	7.55± 1.17 ^a^	8± 1.14	0.04
**Good sleep quality**	68 (87.2%)	25 (59.5%)	51 (63%)	<0.001
**Snoring**	16 (20.51%)	7 (16.66%)	11 (13.6%)	0.506
**Time of dinner^#^**	20:15	20:52	21:06	0.670
**Midnight snacking**	3 (3.8%)	5 (11.9%)	11 (13.6%)	0.052
**Time of sleep^#^**	21:06	21:13	21:20	0.602

Data depicted as number (percentage); mean± std deviation.

# Time presented in a 24-hour clock format.

**Table 3:** Univariate and multiple logistic regression to find the potential association of diabetes risk and chronotypes.

**Variable**	**Univariate Logistic Regression**			**Multiple Logistic Regression**		
	**Crude OR**	**95% CI**	**P-value**	**Adjusted OR**	**95% CI**	**P-value**
Age (per year)	1.05	(1.02-1.08)	**0.001**	1.04	(1.01-1.07)	**0.012**
Gender (Male *vs.* Female)	1.30	(0.85-1.98)	0.221	1.25	(0.82-1.91)	0.298
TC(mg/dL)	1.01	(1.00-1.02)	**0.042**	1.01	(1.00-1.02)	0.052
HDL (mg/dL)	0.96	(0.93-0.99)	**0.010**	0.97	(0.94-1.00)	0.064
LDL (mg/dL)	1.01	(1.00-1.02)	**0.020**	1.01	(1.00-1.02)	**0.038**
VLDL (mg/dL)	1.03	(1.01-1.05)	**0.002**	1.02	(1.00-1.04)	**0.026**
TG (mg/dL)	1.02	(1.01-1.03)	**<0.001**	1.01	(1.00-1.02)	**0.008**
BMI (per kg/m²)	1.15	(1.09-1.21)	**<0.001**	1.12	(1.06-1.18)	**<0.001**
Waist-Hip Ratio	1.50	(1.25-1.80)	**<0.001**	1.40	(1.16-1.69)	**<0.001**
MEQ Chronotype						
Intermediate *vs.* Morning	1.30	(0.90-1.88)	0.165	1.25	(0.86-1.82)	0.245
Evening *vs.* Morning	2.10	(1.45-3.05)	**<0.001**	1.90	(1.30-2.78)	**0.001**
Duration of DM (years)	1.10	(1.05-1.15)	**<0.001**	1.07	(1.02-1.12)	**0.006**
FBS (per mg/dL)	1.03	(1.02-1.04)	**<0.001**	1.02	(1.01-1.03)	**<0.001**
PPBS (per mg/dL)	1.12	(1.06-1.15)	**<0.001**	1.06	(1.03-1.13)	**<0.001**
HbA1c	1.30	(1.18-1.43)	**<0.001**	1.25	(1.13-1.38)	**<0.001**
Sleep Quality						
Moderate *vs.* Good	1.60	(1.10-2.33)	**0.014**	1.45	(1.00-2.10)	**0.049**
Poor *vs.* Good	2.40	(1.55-3.72)	**<0.001**	2.10	(1.35-3.27)	**0.001**
Snoring (Yes *vs.* No)	1.90	(1.22-2.96)	**0.004**	1.75	(1.12-2.74)	**0.015**
Midnight Snacking						
Seldom *vs.* No	1.50	(0.95-2.36)	0.081	1.40	(0.88-2.22)	0.156
Yes *vs.* No	2.20	(1.42-3.41)	**<0.001**	1.95	(1.25-3.05)	**0.003**
Sleep Duration (hours)	0.85	(0.76-0.95)	**0.005**	0.88	(0.79-0.98)	**0.022**
Time of Dinner	1.80	(1.25-2.60)	**0.002**	1.60	(1.10-2.33)	**0.014**
Time of Sleep	1.70	(1.18-2.45)	**0.005**	1.55	(1.07-2.25)	**0.021**

HbA1C: Glycated Haemoglobin; FBS: Fasting blood sugar; PPBS: Postprandial blood sugar; MEQ: Morningness-Eveningness Questionnaire; BMI: Body mass index; TG: Triglyceride; TC: Total cholesterol; LDL: low-density lipoprotein; HDL: High-density lipoprotein; VLDL: very low-density low protein.

## Data Availability

The authors confirm that the data supporting the findings of this research are available within the article.

## LIST OF ABBREVIATIONS

ANOVA: Analysis of Variance
BMI: Body Mass Index
DCFs: Data Collection Forms
DM: Diabetes Mellitus
FBS: Fasting Blood Sugar
HbA1c: Glycated Hemoglobin
HDL-C: High-Density Lipoprotein Cholesterol
hs-CRP: High-Sensitivity C-Reactive Protein
ICF: Informed Consent Form
IEC: Institutional Ethics Committee
LDL-C: Low-Density Lipoprotein Cholesterol
MEQ: Morningness-Eveningness Questionnaire
MVPA: Moderate to Vigorous Physical Activity
PPBS: Postprandial Blood Sugar
SD: Standard Deviation
SPSS: Statistical Package for the Social Sciences
STROBE: Strengthening the Reporting of Observational Studies in Epidemiology
T1DM: Type 1 Diabetes Mellitus
T2DM: Type 2 Diabetes Mellitus
TC: Total Cholesterol
TG: Triglycerides
VLDL-C: Very Low-Density Lipoprotein Cholesterol
WC: Waist Circumference
WHR: Waist-to-Hip Ratio

## STANDARDS OF REPORTING

STROBE guidelines were followed.
